# *Citizen Sensors* for SHM: Use of Accelerometer Data from Smartphones

**DOI:** 10.3390/s150202980

**Published:** 2015-01-29

**Authors:** Maria Feng, Yoshio Fukuda, Masato Mizuta, Ekin Ozer

**Affiliations:** Department of Civil Engineering and Engineering Mechanics, Columbia University, 500 W 120th St, New York, NY 10027, USA; E-Mails: mfeng@columbia.edu (M.F.); yf2290@columbia.edu (Y.F.); mizuta.masato@gmail.com (M.M.)

**Keywords:** smartphone sensors, *Citizen Sensors*, accelerometers, structural health monitoring, post-event damage assessment, civil engineering structures, modal identification

## Abstract

Ubiquitous smartphones have created a significant opportunity to form a low-cost wireless *Citizen Sensor* network and produce *big data* for monitoring structural integrity and safety under operational and extreme loads. Such data are particularly useful for rapid assessment of structural damage in a large urban setting after a major event such as an earthquake. This study explores the utilization of smartphone accelerometers for measuring structural vibration, from which structural health and post-event damage can be diagnosed. Widely available smartphones are tested under sinusoidal wave excitations with frequencies in the range relevant to civil engineering structures. Large-scale seismic shaking table tests, observing input ground motion and response of a structural model, are carried out to evaluate the accuracy of smartphone accelerometers under operational, white-noise and earthquake excitations of different intensity. Finally, the smartphone accelerometers are tested on a dynamically loaded bridge. The extensive experiments show satisfactory agreements between the reference and smartphone sensor measurements in both time and frequency domains, demonstrating the capability of the smartphone sensors to measure structural responses ranging from low-amplitude ambient vibration to high-amplitude seismic response. Encouraged by the results of this study, the authors are developing a citizen-engaging and data-analytics crowdsourcing platform towards a smartphone-based *Citizen Sensor* network for structural health monitoring and post-event damage assessment applications.

## 1. Introduction

With the rapid advances in computer and sensor technologies in the last two decades, structural health monitoring (SHM), mostly based on structural vibration, has become an important research field in civil engineering [1,2]. Implementation of SHM in civil engineering structures, however, has practical difficulties and financial burdens associated with instrumentation and monitoring. Conventional sensors have high hardware, installation, and maintenance costs, as well as remote monitoring and cabling issues. Although wireless sensors aim to solve some of these issues, additional issues are then introduced regarding power consumption, data acquisition and networking. These issues have hindered practical implementation of SHM methodologies on massive scales such as networks of highway bridges and urban areas with large stocks of buildings. To address these issues, many emerging sensor technologies are being developed, including those proposed by the authors' team (e.g., [3,4]).

On the other hand, the Internet, smartphones, and mobile networks have given rise to citizen participation for crowdsourcing applications and producing valuable data. A number of seismology and earthquake engineering projects have shown the benefits of such data. In Southern California, citizens reported experiences to a seismology network after the 1999 7.1-magnitude Hector Mine Earthquake, taking part in mapping the intensity of the earthquake in a project called ShakeMap [5]. “Did you feel it?” the online seismic intensity database, received more than 750,000 responses by 2007, and was able to generate intensity maps in an automated fashion [6,7]. “The Quake Catcher Network” introduced a rapidly deployable seismic network that aimed to increase the number of seismic stations extensively with minimal cost based on inexpensive MEMS sensors and volunteers [8–11]. “Community Seismic Network” is a seismic network which is supported by low-cost accelerometers connected to personal computers or sensors embedded in mobile devices, and uses data fusion techniques to distinguish earthquake-induced vibrations from false alarms [12–14]. “iShake” is the proposed framework for using smartphones as seismographs, and studies [15–17] investigated the reliability of ground motion data obtained from the smartphone sensors. “Community Seismic Network” and “The Quake Catcher Network” are utilized to simulate structural response based on the Timoshenko beam theory [18]. These studies show the potential of using smartphones to measure vibrations.

Encouraged by the recent development and the enthusiasm of citizens to participate, the authors propose a smartphone-based *Citizen Sensor* network to collect structural integrity data at low cost. This network enables a crowdsourcing platform where smartphones act as mobile sensors and provide structural vibration data (pre-processed by the phones) and GPS location data to a cloud server. The long-term vibration measurement data and the subsequently identified structural vibration characteristics will establish a baseline database for the structure for the purposes of structural health monitoring and damage detection, as demonstrated in prior research (which is beyond the scope of this paper). Engaging the crowd will allow efficient monitoring of a large number of structures in an urban setting, which can be particularly useful for rapid assessment of structural damage of buildings and urban infrastructure after a major event such as an earthquake.

This paper represents the first step toward the envisioned *Citizen Sensor* network by investigating the feasibility of using smartphone accelerometers to monitor structural vibration under normal and extreme loads. A number of shaking table tests are conducted to compare smartphone sensor performance with high-quality accelerometers for measuring vibration of different frequencies. Furthermore, input ground motion and response of a column model, subjected to operational loads, white noise and earthquake excitations throughout shaking table tests, are monitored using smartphone and high-quality accelerometers. Finally the smartphone sensor was used to measure ambient and forced vibrations of a bridge.

It is noted that the coupling between the smartphone and the structure can affect the vibration measurement [19]. In this study, the smartphones are fixed on the structures using double-sided tapes to ensure that no local vibration would affect the quality of the structural vibration measurement. In reality, smartphone users would need to place their phones on rigid holders that are permanently fixed on building floors or columns/walls while taking the vibration measurement. The measurement could be automatically triggered by an event (such as an earthquake) when the phones are placed in such holders.

## 2. Smartphone Sensor Properties

The most widely used brands and generations of smartphones, referred to as Smartphone 1, Smartphone 2 and Smartphone 3, are tested in this study. They were carefully selected in order to consider the factors that might have an influence on the smartphone sensors' measurement performance. These factors could be related to hardware such as the accelerometer and processor embedded in the phone, as well as the material and geometrical properties of the phone case. A detailed study on the effects of such physical properties on smartphone seismometer data quality was conducted by applying different coupling conditions and can be found in [15,17]. Software including the measurement application and the phone's operating system might also affect the measurement performance.

Over the last few years, smartphone technology has made significant advances. The phone central processing unit (CPU) and random-access memory (RAM) capabilities have increased significantly while the phone size and weight have decreased [20]. Furthermore, Smartphone 1 and Smartphone 2, two generations of the same smartphone, are embedded with different microelectro-mechanical systems (MEMS) accelerometers, the LIS331DLH [21] and LIS331DL [22], respectively. The accelerometer properties are listed in Table 1, in comparison with high-quality piezoelectric sensors used in this study as reference sensors [23]. In addition, another widely available new generation smartphone, Smartphone 3, was also tested [24].

Several available smartphone applications were tested and the “Seismometer” application was chosen for the vibration measurements in this study. Due to the limitations of the application, the sampling rate is set to 100 Hz for both smartphone sensors, leading to a Nyquist frequency of 50 Hz.

## 3. Small-Scale Shaking Table Tests

**Figure 1.**
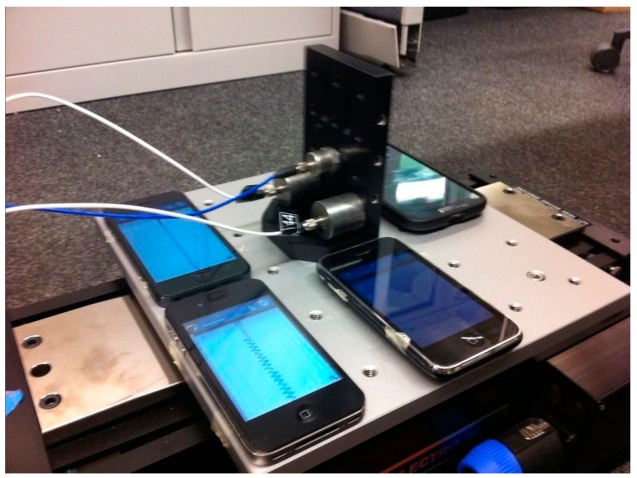
Sinusoidal wave shaking table test setup.

Although sensor datasheets provide extensive information regarding smartphone accelerometers, accelerometers' performance can be influenced by a number of external effects such as phone hardware, embedded filters, and phone geometry. In other words, bare accelerometer performance might be different than an accelerometer embedded in a smartphone. Therefore, in order to investigate the smartphone sensors' capabilities of measuring vibration of different frequencies and amplitudes, small-scale shaking table tests are carried out. As shown in Figure 1, smartphone sensors are fixed on an electromagnetic shaking table, together with two of the high-quality piezoelectric reference accelerometers.

The shaking table is excited with sinusoidal motions of different frequencies including 0.5, 0.8, 1, 2, 5, 10 and 20 Hz. Due to the limitations of the shaking table, low-frequency content sinusoidal wave amplitudes are relatively small compared with high-frequency content sinusoidal waves. As a result, the maximum acceleration amplitudes range from 0.05 g to 0.2 g.

**Figure 2.**
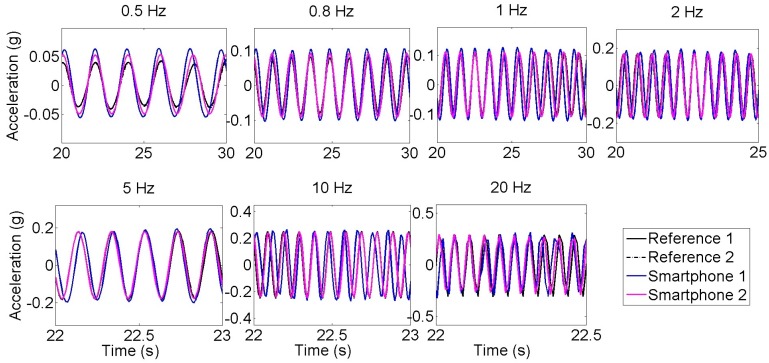
Time history of reference and smartphone sensors under different frequencies.

Figure 2 illustrates the acceleration time histories measured by the reference and smartphone accelerometers under sinusoidal excitations of different frequencies. The measured peak amplitudes by the smartphone sensors agree well with those by the reference sensors, although the smartphone sensors tend to slightly overestimate the amplitude (which is in correlation with Arias Intensity results presented in [17]). It is noted that the reference and the smartphone sensor data are acquired by different data acquisition systems and thus not perfectly synchronized. There are slight differences among the clocks in the smartphones and in the reference sensor data acquisition system, resulting in the slight phase differences in the measured acceleration time histories.

Table 2 summarizes the frequency and the amplitude errors between the reference and the smartphone sensors. It is observed that the new generation smartphone (Smartphone 2) is significantly more accurate than the old generation smartphone (Smartphone 1). For instance, for the 1 Hz excitation, the error between peak horizontal accelerations decreases from 17.5% (Smartphone 1) to 3.10% (Smartphone 2). Similarly, new smartphone sensors are capable of obtaining the dominant frequency of the signal with an error up to 0.96% whereas old generation smartphone errors are significantly large, ranging between 4% and 5%. Although accuracy in frequency slightly changes with different tests, the accuracy in amplitude decreases as peak horizontal acceleration decreases. In conclusion, the error results in Table 2 show that the new generation smartphone (Smartphone 2) is reasonably accurate for measuring vibration in the frequency range relevant to most of the civil engineering structures.

## 4. Large-Scale Seismic Shaking Table Tests of a Structural Model

**Figure 3.**
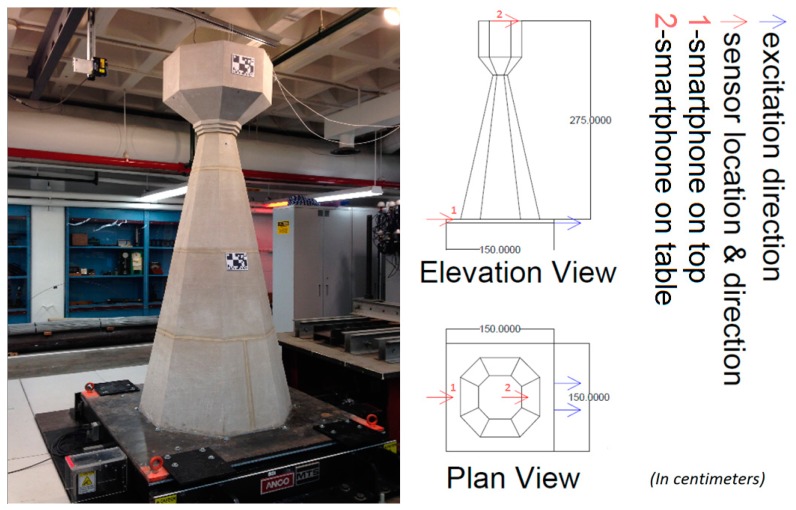
Masonry column model and shaking table.

In order to examine the capabilities of smartphone sensors for measuring different types of vibration at different amplitudes, large-scale seismic shaking table tests are performed on a masonry column model, as shown in Figure 3, involving operational, white noise and earthquake excitation inputs. Further details about the experiment can be found in [25,26]. The smartphone and reference accelerometers are installed on the top of the model, while another smartphone is installed on the top of the shaking table near the foot of the model. The visual inspections before and after the tests show no crack or other types of damage, and thus the structure is assumed to be a linear time invariant system throughout the tests. In previous studies, the same assumption is used by the authors in [27,28], and the crack-damage relationship can be observed from the shaking table test data as shown in [29,30]. The vibration measurements are used to identify modal characteristics of the structure. In order to determine modal frequencies, power spectral densities are used. Prior to computation of power spectral densities, operational, white-noise, and earthquake excitation test time histories are subjected to zero-padding to smoothen the spectral curves. Therefore, actual spectral resolutions, 0.0100, 0.0142, and 0.0142 Hz, respectively, are converted into 0.0015 Hz as a result of zero-padding the original time signals.

### 4.1. Operational Vibration Tests

**Figure 4.**
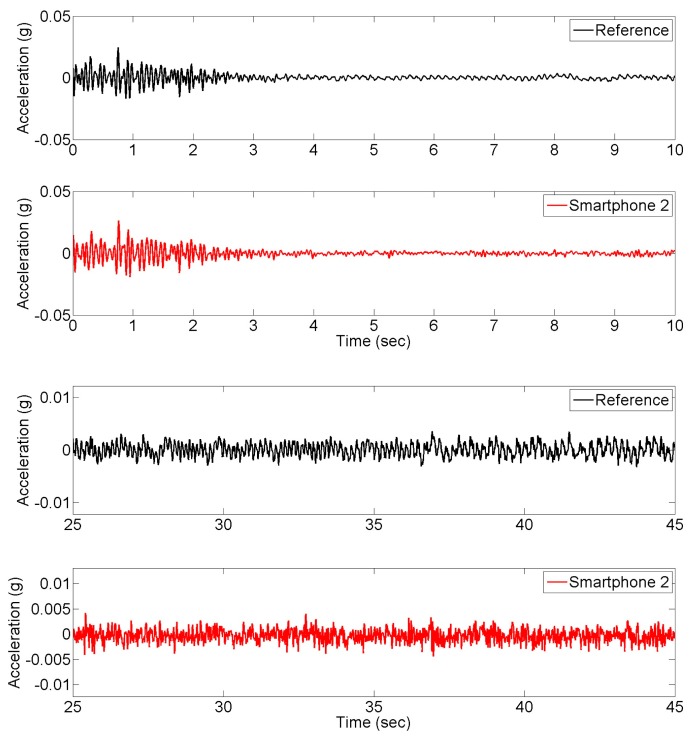
Time history of impact and operational vibration response measurements.

**Figure 5.**
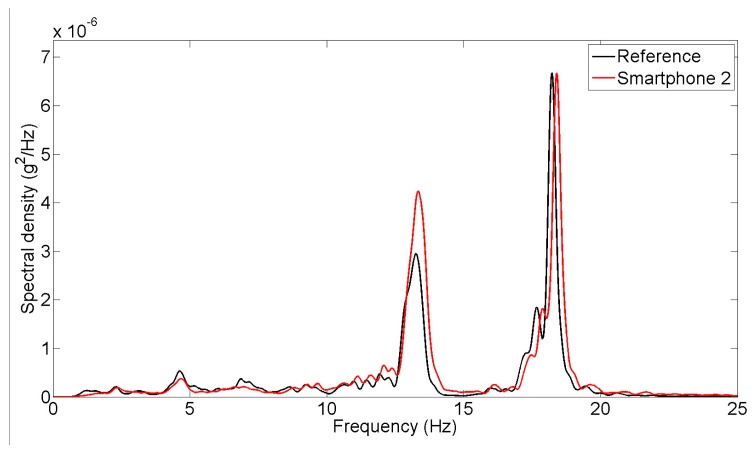
Power spectral density of impact and operational vibration response measurements.

First, the seismic shaking table is locked and the responses of the column model to environmental vibrations and to hammer impact loading on top are measured. Figure 4 shows the acceleration time history responses measured at the top of the column by the reference and smartphone sensors under the hammer impact loading and under the operational vibrations respectively. The plots corresponding to reference and smartphone sensors are plotted separately because that the measurements are not synchronized, yet the time histories show the similarities between the two measurements. Likewise, error is not quantified as a function of time since samples obtained from reference and smartphone sensors refer to different time instants. Cross-correlation or GPS synchronization can be addressed to deal with this problem, which can be addressed in the future. The peak response to the impact load is approximately 0.02 g, while the operational vibration amplitude is less than 0.004 g. It is observed that smartphone measurements agree well with the reference measurements in terms of amplitude characteristics. Figure 5 shows the power spectral densities of the vibration responses measured by the reference and smartphone sensors, demonstrating frequency characteristics of the measured responses are significantly close to each other. In other words, Figure 5 reflects the spectra of the response to the initial impact followed by operational vibrations.

### 4.2. White-Noise Excitation Tests

**Figure 6.**
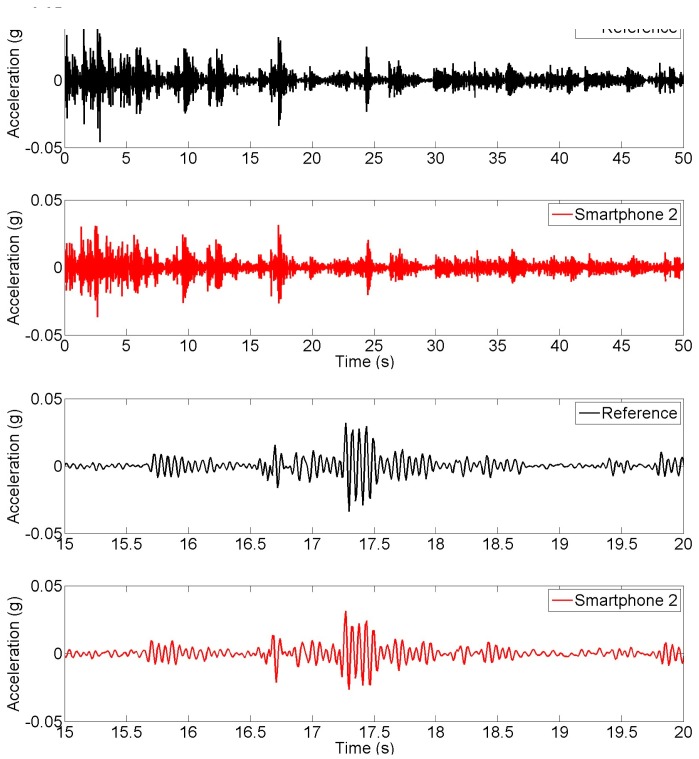
Time history of white noise excitation response measurements.

**Figure 7.**
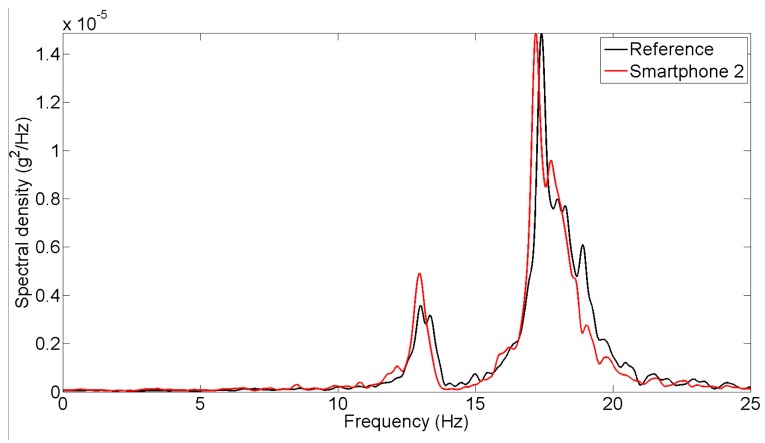
Spectral density of white noise excitation response measurements.

The seismic shaking table is excited by white-noise ground motion input and the smartphone and reference sensors measure the response of the column model. Figure 6 compares the time history responses obtained from the reference and smartphone sensors. Figure 7 shows the power spectral densities of reference and smartphone measurements. Significant agreement is observed in both the time and frequency domains.

### 4.3. Earthquake Excitation Tests

**Figure 8.**
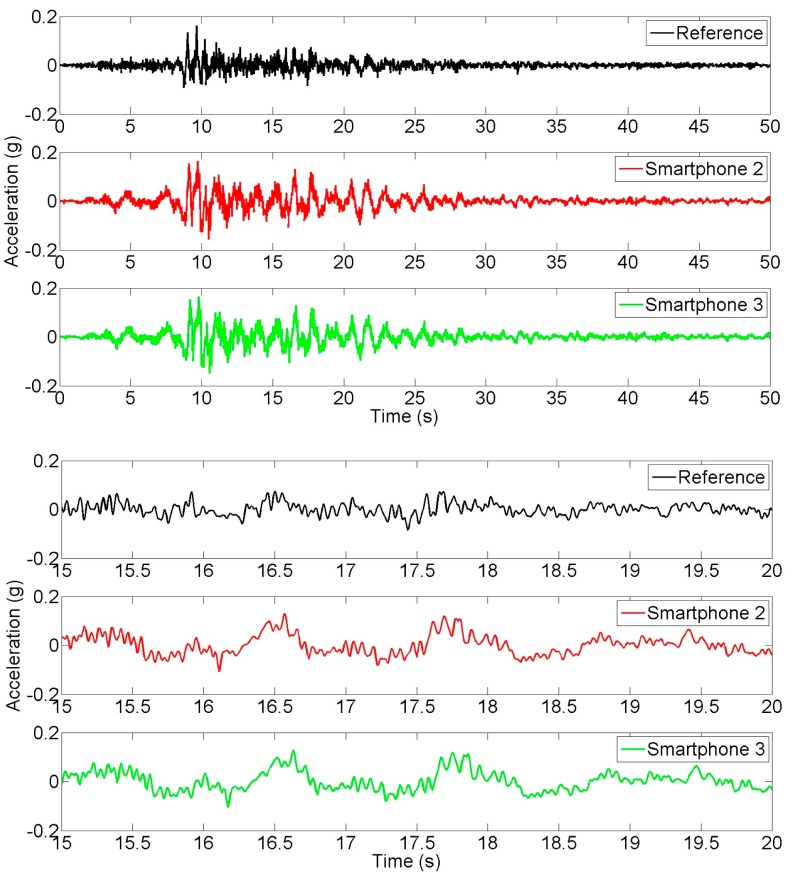
Time history of targeted and achieved earthquake input ground motion measurements.

**Figure 9.**
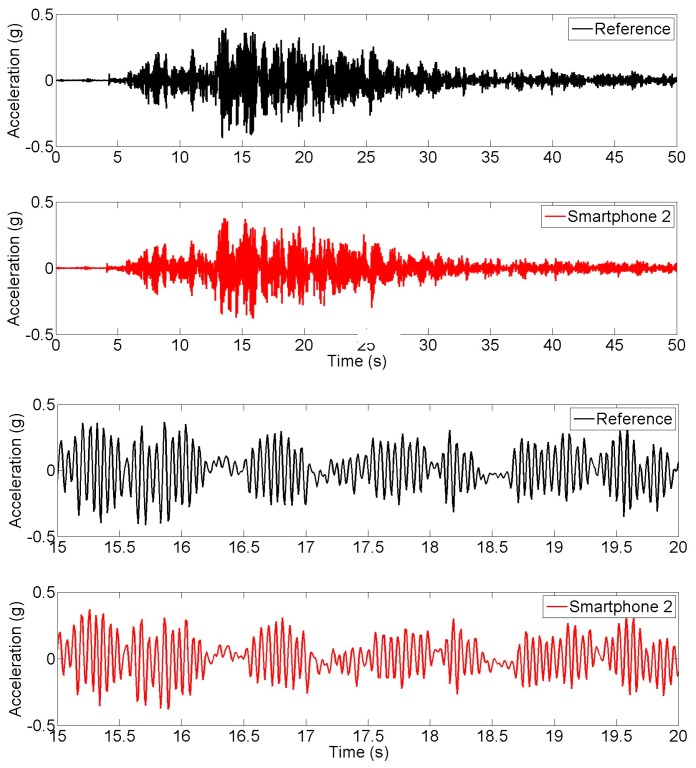
Time history of earthquake response measurements.

Finally, seismic input ground motion and response measurements are made in order to evaluate the smartphone performance in measuring seismic strong motion and structural response. Figure 8 show the input ground motion time histories targeted by shaking table controller (Reference), and the ones measured by Smartphone 2 and Smartphone 3. The bottom three plots in the figure are the enlarged portions (between 15 s and 20 s) to show more details. It is noted that the shaking table acceleration (the input) was not measured by the reference sensor. The “Reference” in Figure 8 refers to the input seismic acceleration generated by the controller of the seismic shaking table. The reference sensor was used for measuring the structural response in this seismic excitation experiment. An excellent agreement is observed between the measurements made by the two different smartphones. A considerable difference is observed between the target time history and the measurements by the smartphone sensors, due to the fact that a seismic shaking table has physical limitations of generating targeted motion [31]. Figure 9 shows the acceleration response time histories measured at the top of the model by the reference sensor and Smartphone 2. Similarly, portions of the top two plots are enlarged in the bottom two plots to show more details. An excellent agreement is observed between the responses measured by the smartphone and the reference sensor.

**Figure 10.**
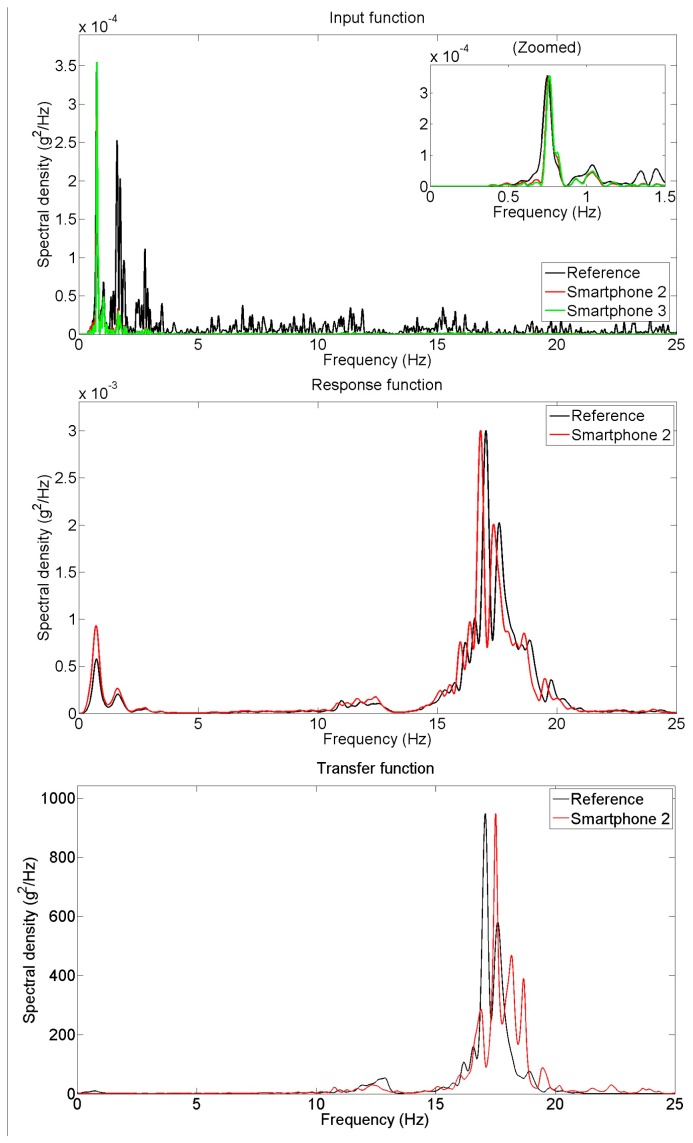
Spectral density of earthquake excitation measurements.

Power spectral densities are obtained from the targeted input, measured input and response acceleration time histories and plotted in Figure 10, based on which the transfer function is developed and plotted in the same figure. Again, the spectral densities of the responses measured by the reference and the smartphone sensors agree well. Although the ground motions of two different smartphones have significant difference with the target input motion applied to the shaking table, they agree very well in the frequency domain as well.

### 4.4. Comparison of Identified Natural Frequencies of the Structural Model

Natural frequencies of the masonry column model are identified based on the measurements made in the seismic shaking table tests involving the different types of excitations. The peak picking method is applied to extract the natural frequencies from the power spectral densities of the response acceleration under the operational and white-noise excitations shown in Figures 5 and 7. For the seismic excitation, the natural frequencies are identified from the spectral density function plot in Figure 10. The identified fundamental frequency values are summarized in Table 3. From the measurements made by the reference sensor, the fundamental frequency of the column model is identified as 18.2, 17.4, and 17.1 Hz respectively under the operational, white noise, and earthquake excitations. Their counterparts measured by the smartphone sensor are 18.4, 17.2, and 17.5 Hz. The frequency values measured by the smartphone sensor and the reference sensors are highly comparable, demonstrating the capability of the smartphone sensor in measuring a structure's natural frequencies.

Furthermore, it is observed that the fundamental frequency of the structural model decreases as its vibration amplitude increases. This phenomenon has been confirmed by many other studies [32–34] and further analysis is beyond the scope of this paper.

## 5. Field Tests of a Bridge

**Figure 11.**
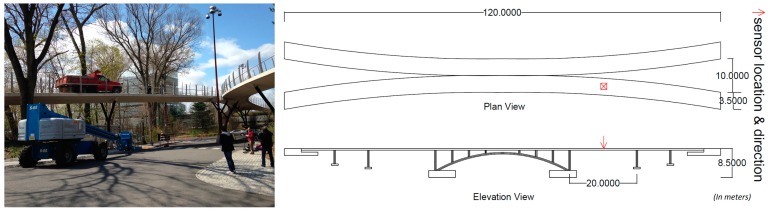
Pedestrian bridge in Princeton (NJ, USA).

In order to investigate the performance of smartphone sensors on actual structures, a series of field tests are conducted on a pre-stressed reinforced concrete pedestrian bridge located in Princeton (NJ, USA) shown in Figure 11. Smartphone 2 and the reference sensor are fixed by double-sided adhesive tapes in the mid span of the bridge deck to measure its ambient vibration and response to dynamic loading. Two sets of dynamic loading tests are carried out. First, a group of participants runs randomly on the bridge with different speeds, rhythms and directions to generate dynamic loads of broader frequency content. Second the same group jumps synchronically at 3 Hz, which is close to the estimated natural frequency of the bridge, to excite the first mode of vibration. Similar to the previous tests, in ambient vibration, random dynamic, and synchronized dynamic test time histories, zero-padding is applied. Therefore, actual spectral resolutions, 0.0142, 0.0067, and 0.0033 Hz, respectively, are converted into 0.0015 Hz as a result.

### 5.1. Ambient Vibration Test

**Figure 12.**
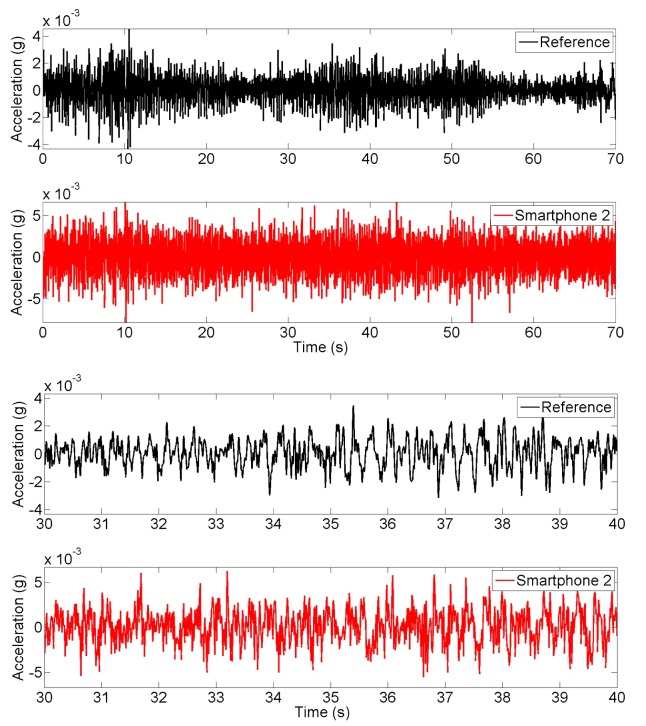
Time history of ambient vibration response measurements.

**Figure 13.**
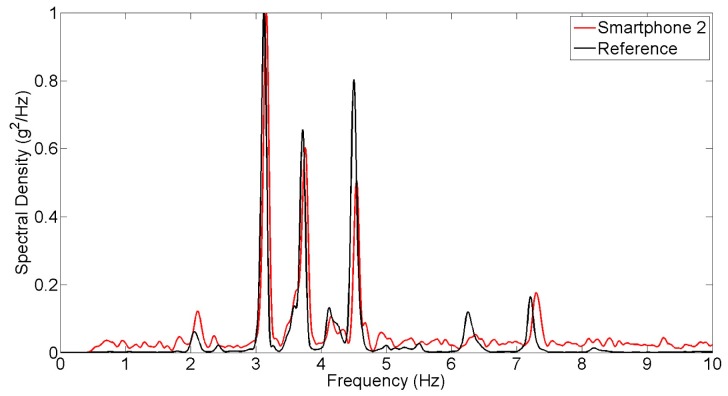
Spectral density of ambient vibration measurements.

Ambient vibration of the bridge, resulting from the environmental vibration caused by pedestrians and vehicles passing under the bridge, are measured using the smartphone and reference accelerometers located at the mid span. Figure 12 compares the time histories obtained from the reference and smartphone accelerometers. The bottom two plots are enlarged portions to show more details. First, the amplitude of the vibration is less than 0.005 g. At this low amplitude, the smartphone sensor is not as sensitive as the high-quality reference sensor, and as a result some differences between the two measurements are observed in the time histories. However, the frequency domain characteristics measured the two sensors match quite well, as shown in the power spectral density plots in Figure 13. For example, the fundamental frequency of the bridge identified from the reference sensor measurement is 3.13 Hz compared with 3.16 Hz by the smartphone measurement. The error is less than 1%. The higher modes by the two measurements also agree well. Moreover, measurements include smartphone sensors positioned without fixing, which also resulted in the same accuracy. In other words, the smartphone sensors are free to move on the structure, yet coupled by the friction between the phone surface and the bridge surface. This implies the practicality of smartphone sensors for vibration measurement. A detailed study, considering different coupling conditions and targeting the effect of fixity on smartphone sensors as seismic instruments is conducted in [15,17].

### 5.2. Random Dynamic Test

**Figure 14.**
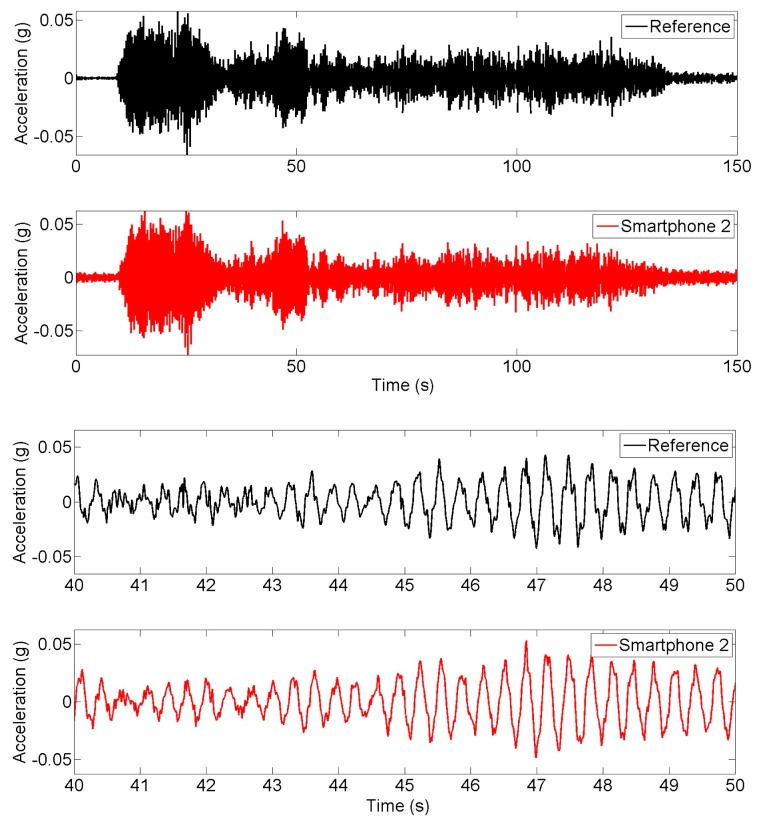
Time history of random dynamic test measurements.

**Figure 15.**
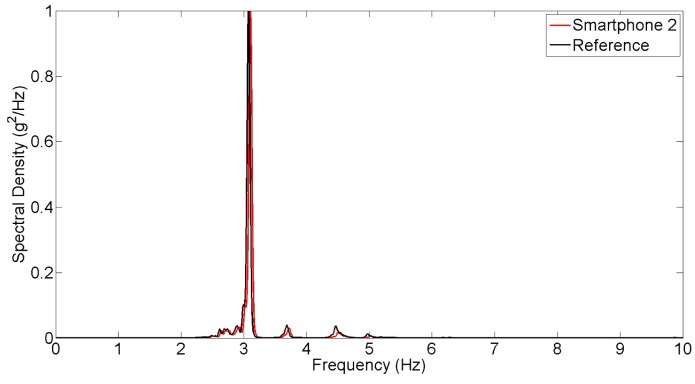
Spectral density of random dynamic test measurements.

In order to apply dynamic loads with broadband frequency content to the bridge, a group of pedestrians run on the bridge deck randomly with different, varying speeds, rhythms and directions without any particular pattern. Figure 14 shows that the smartphone measurement agrees much better with the reference sensor measurement than it does in the ambient vibration case (shown in Figure 13). This is because of the increased vibration amplitude; in fact the random running-induced vibration is ten times higher than the ambient vibration. From the power spectral density plots in Figure 15, natural frequency of bridge is estimated as 3.08 Hz and 3.11 Hz respectively from reference and smartphone sensor measurements, resulting in an error less than 1%.

### 5.3. Synchronized Dynamic Test

**Figure 16.**
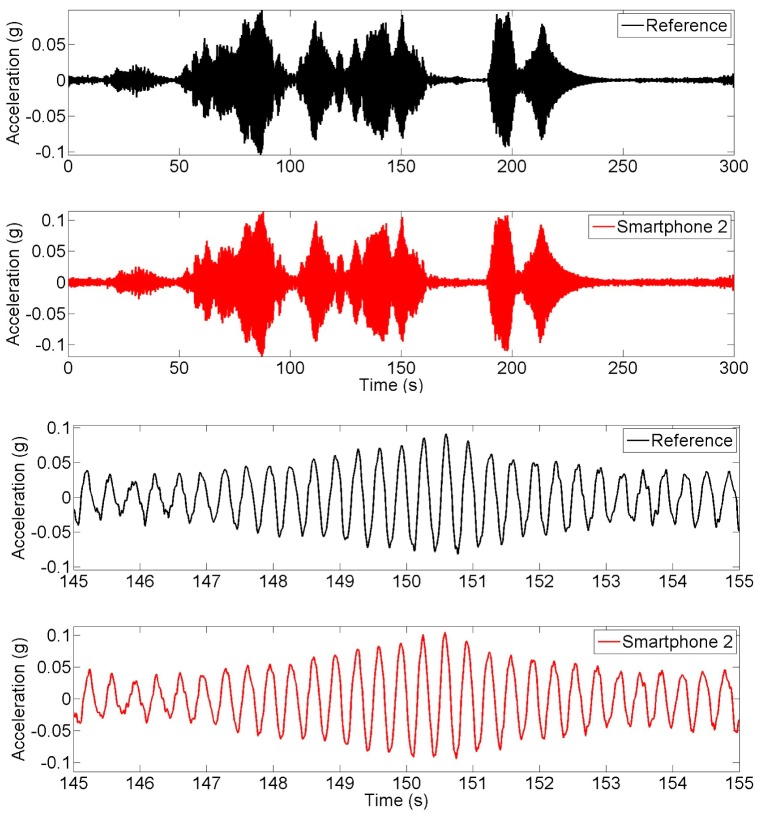
Time history of synchronized dynamic test measurements.

**Figure 17.**
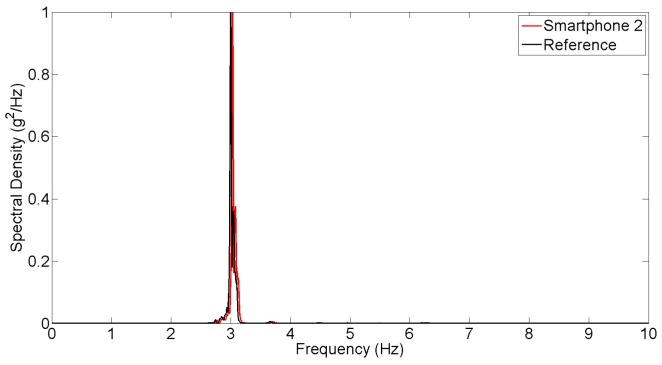
Spectral density of synchronized dynamic test measurements.

Finally, in order to maximize the dynamic load effect, the pedestrian participants jump on the mid span of the bridge deck synchronically at a frequency of 3 Hz, which is close to the estimated natural frequency of the bridge. Figure 16 plots the time histories obtained from the reference and smartphone accelerometers. Due to the dynamic amplification, the bridge response acceleration exceeds 0.1 g. As the vibration amplitude increases, the measurement error of smartphone sensor (with respect to the reference sensor) becomes insignificant. The power spectral densities based on the smartphone and reference measurements, as plotted shown in Figure 17, show their excellent agreement. This synchronized jumping excited only the first mode, which is 3.00 Hz (by the reference sensor) and 3.03 Hz (by the smartphone sensor). Likewise, the error is less than 1%.

### 5.4. Comparison of Identified Natural Frequencies of the Bridge

Natural frequencies of the bridge are identified based on the measurements made in the field tests. The peak picking method is applied to extract the natural frequencies from the power spectral densities of the response acceleration under the ambient, random and synchronized excitations shown in Figures 13, 15 and 17. Table 4 compares the identified fundamental frequency values. From the measurements made by the reference sensor, the fundamental frequency of the bridge is identified as 3.13, 3.08, and 3.00 Hz respectively under the ambient vibration, and the random and synchronized dynamic loading tests, while their counterparts made by the smartphone sensor are 3.16, 3.11, and 3.03 Hz. Again, frequency values measured by the smartphone sensor and the reference sensors are highly comparable, demonstrating the capability of the smartphone sensor in measuring a structure's natural frequencies. Like the observation made in the seismic shaking table tests, the fundamental frequency of the bridge decreases as its vibration amplitude increases.

## 6. Conclusions

A comprehensive experimental study, involving seismic shaking table tests and bridge field tests, was carried out to investigate the performance of smartphone accelerometers in measuring structural response to dynamic loading ranging from low-amplitude ambient to high-amplitude seismic excitations, as well as sinusoidal excitations. Three widely-used smartphones embedded with different accelerometers and a high-quality reference sensor are tested on a small shaking table, a structural model on a large seismic shaking table, and an actual bridge. All the measurement results are compared in both time and frequency domains. The following conclusions can be drawn from this study:

(1) The small-scale shaking table tests confirm that the smartphone sensors are capable of accurately measuring sinusoidal vibration of 0.5 Hz through 20 Hz, a frequency range relevant to most civil engineering structures. The measurement error in terms of the vibration amplitude, when compared with the high-quality reference sensor, is less than 5% for vibration higher than 1 Hz, but increases as the peak horizontal acceleration decreases. The measurement error in terms of vibration frequency is 1% and 5% respectively for the new and the old generation smartphone sensors.
(2) The large-scale seismic shaking table tests of the structural model and the field dynamic tests of the bridge demonstrate the capabilities of smartphone sensors in measuring structural responses to a variety of dynamic loads of different amplitude as well as frequency characteristics. Despite the measurement error of the structural response in the time domain under the low-amplitude (less than 0.005 g) ambient vibration, it is possible to extract the structures' fundamental frequencies with remarkably small error of less than 1%.
(3) The two types of the widely-used smartphones with different operating systems and different accelerometers show comparable performance. The accelerometer in the newer generation smartphone is significantly more accurate than that in the old generation smartphone. The quality of the sensors embedded in smartphones is expected to continue to improve in the future.
(4) The laboratory and field tests show advantages of the smartphone sensors over the conventional sensor, such as the ease of installation and data acquisition as well as wireless transmission.

It is noted that many issues are yet to be solved such as the on-phone signal pre-processing, power-efficient signal transmission and practical phone-structure couplings. Nevertheless, this study demonstrates the feasibility of using smartphone accelerometers for measurement of structural vibration characteristics, from which structural health can be diagnosed as shown in prior research. Encouraged by the results of this study, the authors are exploring the potential of forming a smartphone-based low-cost *Citizen Sensor* network for structural health monitoring and post-event damage assessment in structure- and city-scales, by developing frameworks of citizen engagement, online database and crowdsourcing data analytics.

## Figures and Tables

**Table 1.** Reference and smartphone sensor properties.

**Property**	**Reference**	**Smartphone 1**	**Smartphone 2**	**Smartphone 3**
Sensor maker	PCB Piezotronics	ST Micro-electronics	ST Micro-electronics	Bosch Sensortec
Sensor model	393B04	LIS331DL	LIS331DLH	SMB380
Phone maker, model & Operating System (OS)/Data Acquisition (DAQ) model	NI SCXI-1531	iPhone 3GS, iOS	iPhone 5, iOS	Samsung Galaxy S4, Android
Type	Piezoelectric	MEMS	MEMS	MEMS
Sensitivity ± 2 g	1000 mV/g	18 mg/digit	1 mg/digit	3.9 mg/digit
Measurement range	5 g	2 g	2 g	2 g
Output data rate/Frequency range	0.05–750	100, 400	0.5–1000	3000
Noise density $\left(mg\sqrt{Hz}\right)$	0.00004	N/A	0.218	0.5

**Table 2.** Peak horizontal acceleration error between smartphone and reference accelerometers.

**Frequency (Hz)**		**0.5**	**0.8**	**1**	**2**	**5**	**10**	**20**
Error (%)	Smartphone 1	4.57	4.58	5.04	5.03	4.73	4.96	4.01
	Smartphone 2	0.92	0.95	0.92	0.92	0.95	0.92	0.96

Amplitude (g)		0.04	0.08	0.11	0.17	0.18	0.25	0.29

Error (%)	Smartphone 1	43.9	25.6	17.5	8.19	15.3	17.3	25.7
	Smartphone 2	17.4	8.51	3.10	4.97	1.14	0.45	3.82

**Table 3.** Comparison of structure's natural frequencies identified from different measurements.

**Excitation Type**		**Ambient**	**White Noise**	**Earthquake**
Natural frequency (Hz)	Reference	18.2	17.4	17.1
	Smartphone	18.4	17.2	17.5

Error (%)		1.10	1.15	2.34

**Table 4.** Comparison of bridge's natural frequencies identified from different measurements.

**Excitation Type**		**Ambient**	**Random**	**Synch**
Natural frequencies (Hz)	Reference	3.13	3.08	3.00
	Smartphone	3.16	3.11	3.03

Error (%)		0.96	0.97	1.00
